# The Effective Population Size of Malaria Mosquitoes: Large Impact of Vector Control

**DOI:** 10.1371/journal.pgen.1003097

**Published:** 2012-12-13

**Authors:** Giridhar Athrey, Theresa K. Hodges, Michael R. Reddy, Hans J. Overgaard, Abrahan Matias, Frances C. Ridl, Immo Kleinschmidt, Adalgisa Caccone, Michel A. Slotman

**Affiliations:** 1 Department of Entomology, Texas A&M University, College Station, Texas, United States of America; 2 Department of Epidemiology and Public Health, Yale University, New Haven, Connecticut, United States of America; 3 Department of Mathematical Sciences and Technology, Norwegian University of Life Sciences, Ås, Norway; 4 Medical Care Development International, Malabo, Equatorial Guinea; 5 Malaria Research Lead Programme, Medical Research Council, Durban, South Africa; 6 London School of Hygiene and Tropical Medicine, London, United Kingdom; 7 Department of Ecology and Evolutionary Biology, Yale University, New Haven, Connecticut, United States of America; Stanford University, United States of America

## Abstract

Malaria vectors in sub-Saharan Africa have proven themselves very difficult adversaries in the global struggle against malaria. Decades of anti-vector interventions have yielded mixed results—with successful reductions in transmission in some areas and limited impacts in others. These varying successes can be ascribed to a lack of universally effective vector control tools, as well as the development of insecticide resistance in mosquito populations. Understanding the impact of vector control on mosquito populations is crucial for planning new interventions and evaluating existing ones. However, estimates of population size changes in response to control efforts are often inaccurate because of limitations and biases in collection methods. Attempts to evaluate the impact of vector control on mosquito effective population size (*N_e_*) have produced inconclusive results thus far. Therefore, we obtained data for 13–15 microsatellite markers for more than 1,500 mosquitoes representing multiple time points for seven populations of three important vector species—*Anopheles gambiae*, *An. melas*, and *An. moucheti—*in Equatorial Guinea. These populations were exposed to indoor residual spraying or long-lasting insecticidal nets in recent years. For comparison, we also analyzed data from two populations that have no history of organized vector control. We used Approximate Bayesian Computation to reconstruct their demographic history, allowing us to evaluate the impact of these interventions on the effective population size. In six of the seven study populations, vector control had a dramatic impact on the effective population size, reducing *N_e_* between 55%–87%, the exception being a single *An. melas* population. In contrast, the two negative control populations did not experience a reduction in effective population size. This study is the first to conclusively link anti-vector intervention programs in Africa to sharply reduced effective population sizes of malaria vectors.

## Author Summary

The battle against malaria mosquitoes in sub-Saharan Africa is being fought with two main weapons: indoor residual spraying of insecticides (IRS) and long-lasting insecticidal net (LLIN) campaigns. Although many programs have been successful in reducing malaria infection, demonstrating the impact of these programs on vector populations is typically confounded by numerous variables associated with collection methods. Here, we analyzed more than 1,500 samples of three important malaria vectors—*Anopheles gambiae*, *An. melas*, and *An. moucheti*—from seven sites in Equatorial Guinea that were collected over the course of anti-vector programs in that country (2004–2010). Taking advantage of recently developed coalescent genetic approaches, we derived the demographic history, estimated the effective population size, and determined the timings of population size changes. We demonstrate convincingly for the first time that both IRS– and LLIN–based control resulted in lowered effective population sizes in most cases. No such reductions were observed in negative control populations. Our findings are especially important to malaria control because estimating population sizes using conventional trapping approaches is often limited by our ability to obtain representative samples. More importantly, we were able to conclusively link anti-vector interventions to genetic impacts, a linkage that has been difficult to establish in the past.

## Introduction

Throughout much of sub-Saharan Africa, *Anopheles gambiae* s.s. is the most important vector of malaria, a disease that claims over 780,000 lives every year [1]. Its effectiveness as a vector stems from its close association with human habitat, its habit of readily entering houses at night to feed, and its preference for human blood meals. This species is comprised of two molecular forms; the M and S form [2], [3] which are considered incipient species. *An. gambiae* s.s. also has six, morphologically nearly-identical sibling species. Together these make up the *An. gambiae* species complex, also known as *An. gambiae* s.l. One of these, *Anopheles melas*, is a dominant vector in locations along the West African coast, where it breeds within mangrove belts and tidal swamps [4]. On Bioko Island, Equatorial Guinea, *An. melas* feeds readily on humans, both indoors and outdoors [5], and together with *An. gambiae* s.s. is responsible for malaria transmission [6], [7]. Besides the *Anopheles gambiae* complex, various other species contribute to transmission as well, and in the equatorial rainforests of Central Africa the anthropophilic and endophilic *Anopheles moucheti* is an important vector [4].

Malaria vectors have been subjected to insecticide-based anti-vector interventions throughout numerous locations in sub-Saharan Africa. Currently, the two most frequently applied vector control methods are indoor residual spraying (IRS) and long-lasting insecticidal nets (LLINs), which in recent years have largely replaced insecticide-treated nets (ITNs). Both types of approaches have had a demonstrable impact on malaria transmission in a variety of locations throughout sub-Saharan Africa [8], [9]. At least part of this impact is the result of reductions in mosquito abundance, rather than reduced contact between mosquitoes and humans, or reductions in mosquito longevity. Reduced abundance has been reported in several locations in which IRS and ITNs/LLINS have been implemented [eg.], [ 6,10], although not all studies observed a noticeable reduction in mosquito density in areas controlled by ITNs [reviewed in 11].

On occasion, vector control has resulted in the disappearance of a vector species. For example, in the Pare region of Tanzania an indoor residual spraying campaign with dieldrin in the 1950's eliminated *An. funestus* and is estimated to have reduced the abundance of *An. gambiae* s.l. to one-fifth of its former density . After control was eliminated in 1959, it took approximately five years before *An. gambiae* densities approached pre-intervention levels [12]. On Bioko Island, Equatorial Guinea, both *An. funestus* and the S form of *An. gambiae* all but disappeared soon after the start of an IRS campaign in 2004 [6]. In two villages in Kenya, *An. gambiae* s.s. has largely disappeared following the start of ITN/LLIN distribution, although the abundance of its sibling vector species *An. arabiensis* was not impacted [10]. These cases are species and location specific and the result of vector control on abundance depends on the behavior, ecology, and possibly the genetics of a vector species. For example, *An. melas* and *An. gambiae* (M) remain abundant on Bioko Island after the disappearance of *An. funestus* and *An. gambiae* (S) [5].

Except when species are eliminated, estimates of changes in mosquito abundance can be imprecise due to a variety of limitations in trapping approaches. For example, indoor collections may be strongly influenced by the repellent effects of residual insecticides present within the home, or shifts in host choice. Weather conditions can also greatly affect any comparisons of abundance between time points. In the case of window traps, consistent and continuous operation of the trap by the home occupant may be compromised over time. For example, Sharp *et al.* [6] reported virtually no anopheline mosquitoes collected using window traps on Bioko after two years of IRS, even though subsequent light trap catches and human landing catches showed that both *An. melas* and *An. gambiae* (M) were present in large numbers [5, HJ Overgaard et al. unpublished data]. However, it remains to be answered whether these reported decreases in abundance correspond to changes in the genetic size of a population, i.e. the effective population size (*N_e_*), which ultimately reflects on the impact of anti-vector interventions on mosquito populations.

The effective population size is defined as the size of an ideal population that experiences genetic drift, accumulates inbreeding or looses variation at the same rate as the actual population [13], [14]. *N_e_* is central to the dynamics of several population genetic processes, such as migration, drift and selection, all of which determine the amount of genetic variation that is present in a population. Typically, *N_e_* is smaller than the total population (census size), owing to a variety of factors including variance in reproductive success among individuals in a population. Only two studies have assessed changes in effective population size in malaria vectors in response to control measures, but both failed to provide a definitive answer [15], [16].

Wondji *et al.* [16] examined the impact of ITN distribution on the genetic structure of *An. arabiensis* populations in a village in northern Cameroon by estimating *N_e_* both before and after a local ITN distribution campaign. These authors used the temporal method based on the standardized variance in allele frequencies [17], and detected a non-significant decline in *N_e_* following ITN distribution. Furthermore, the decline was transient, which the authors attributed to the small scale of the intervention and the migration of *An. arabiensis* mosquitoes from neighboring populations into the study village.

Pinto *et al.* [15], [18] examined the impact of a successful IRS-based nation-wide malaria control program conducted in the early 1980's in the archipelago of São Tomé and Principe. Even though this project significantly reduced indoor mosquito densities, a microsatellite-based study did not detect any signs of a bottleneck associated with this control effort, calling into question the effectiveness of IRS in reducing malaria vector populations. It was proposed that the outdoor feeding and resting tendencies of the vector on the island [19] might have prevented exposure of the vector to the insecticide. However, these authors used the program Bottleneck [20] for their analysis, which recently shown to have a potential false-negative period spanning 2–4*N_e_* generations [21]. Even *N_e_* estimates of only a few thousand would necessitate a gap of several thousand generations separating the event and sampling, before a bottleneck could reliably be detected. Therefore, despite decades of malaria vector control, no study has ever convincingly demonstrated the impact of ITN/LLIN or IRS interventions on the *N_e_* of malaria vectors.

Applying a rigorous sampling of multiple populations combined with the use of recently developed coalescent-based methods could help us answer this long-standing question. The Approximate Bayesian Computation method [ABC, 22], is a coalescent simulation based approach that compares summary statistics from observed data with data from simulated hypothetical scenarios to determine the most likely demographic scenario. ABC methods are now being widely employed for understanding evolutionary histories of populations [for examples], [ see 23], [24]–[26], for estimating parameters of interest such as the effective population size, or the timing of population size changes. ABC has proven especially valuable in recovering demographic history in a variety of contexts: for example, Lombaert *et al.*, [27] addressed phylogeographic questions and inferred routes of invasion in an invasive ladybird species; Wegmann *et al.*, [28] re-evaluated evolutionary hypotheses about chimpanzee population history and found evidence for a population expansion. Athrey *et al.*, [29] used temporal samples to estimate the timing and magnitude of population declines in the endangered Black-capped Vireo. The ABC approach is especially powerful if genetic samples are collected across several generations, with the accuracy and precision of estimates increasing with the number of generations between samples. Accuracy of estimates is also expected to increase with the use of summary statistics, compared to other estimators that may rely on single variance components.

Here, we determine the extent to which IRS and/or LLIN use in Equatorial Guinea has impacted the effective population sizes of three important malaria vectors; *An. gambiae* (M+S), *An. melas* and *An. moucheti*. Mosquito populations in Equatorial Guinea have been subject to IRS and/or ITNs as part of the Bioko Island Malaria Control Project (BIMCP) on Bioko Island and the Equatorial Guinea Malaria Control Initiative (EGMCI) in continental Equatorial Guinea in cooperation with the National Malaria Control Program. On Bioko Island this has resulted in malaria infection rates in children falling from 42% to 18% after four years of high IRS coverage [30], and from 59% to 46% on the mainland after four years of IRS and LLIN coverage (unpublished data, Medical Care Development International). Our study is based on a series of samples collected between 2004 and 2010, and with the exception of one location, were collected before or concurrent with the start of anti-vector intervention programs in Equatorial Guinea. This temporal sequence empowers us to probe the demographic history of these populations in relation to the impact of vector control measures.

We studied a total of seven Equatoguinean populations – two on Bioko island (Arena Blanca and Punta Europa), and five mainland populations (Cogo, Mongomo, Niefang, Ukomba and Yengue). In addition to these seven study populations, we also analyzed existing microsatellite data from two negative control populations that did not experience vector control –Tiko in Cameroon and Fanzana in Mali. This was done to examine how seasonal or yearly fluctuations in population size affect our inference of demographic history. Our main objectives were to determine if vector control has resulted in lowered effective population sizes of malaria vector mosquitoes. In six out of seven study populations, vector control coincided with a demographic bottleneck that substantially lowered effective population sizes of malaria vectors. In the negative control populations, no reduction in effective population sizes was observed.

## Results

**Figure 1:**
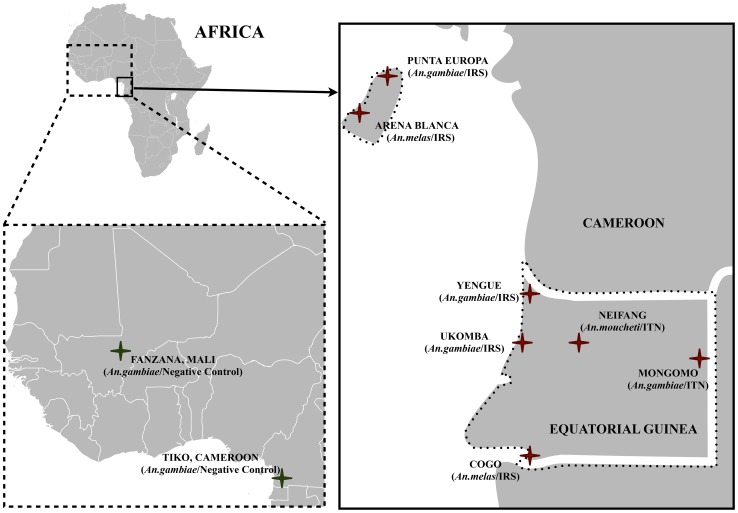
A map indicating the seven study sites in Equatorial Guinea. Two locations were sampled on Bioko Island and five locations on mainland Equatorial Guinea. Additionally, two negative control populations; Tiko in Cameroon and Fanzana in Mali were included in our study.

A data set comprising 13 (*An. moucheti)*, 15 (*An. melas*) or 17 (*An. gambiae*) microsatellite markers was obtained for a total 1,519 mosquitoes from the study populations (Figure 1) from Equatorial Guinea. This data represents 2 to 4 time points for each of the seven populations included in this study (Table 1). After discarding markers showing evidence for the presence of null alleles, the following number of loci were included in our final analyses: *An. gambiae* M form, Punta Europa 13 loci; *An. gambiae* M form, Ukomba 14 loci; *An. gambiae* S form, Mongomo 13 loci; *An. gambiae* S form, Yengue 16 loci; *An. moucheti*, Niefang 13 loci; *An. melas*, Arena Blanca 13 loci, *An. melas*, Cogo 12 loci. For the two negative control populations (Tiko, Cameroon and Fanzana, Mali, Figure 1), microsatellite datasets containing 11 loci were analyzed for a total of 236 samples.

**Table 1:** Study species, sites, sampling years, and sample sizes that were the basis for estimations of *N_e_* and comparisons of alternate demographic scenarios in ABC analysis.

Species	Location	No. of Houses	Intervention Type	Start of Intervention	Sampled Year	Sample Size (N)
***An. gambiae (M)***	Punta Europa	∼80	IRS	2004	April 2004	119
					September 2006	63
					August 2007	78
					April 2010	94
	Ukomba	∼35000	IRS	Late 2007	March 2007	78
					May 2009	46
					Feb 2010	95
***An. gambiae (S)***	Mongomo	∼6000	ITN	Early 2008	Feb 2007	83
					April 2009	56
					May 2010	53
	Yengue	∼50	IRS	Late 2007	Feb 2007	62
					May 2009	92
					May 2010	32
***An. moucheti***	Niefang	∼2000	ITN	Early 2008	May 2007	48
					Aug 2009	34
***An. melas***	Arena Blanca	∼40	IRS	2004	April 2009	89
					Sept 2010	68
	Cogo	∼400	IRS	Late 2007	April 2007	70
					Nov 2007	76
					June 2010	50

Severe bottlenecks are expected to reduce genetic variability and thus reduce both allelic richness (*A_R_*) and heterozygosity (*H_E_*), especially when populations become very small, although *H_E_* is expected to decline at a slower rate compared to *A_R_*. Values for *A_R_* and *H_E_* for each time point in the seven populations are reported in Table 2. Six of the seven sampled populations displayed a decline in *A_R_* between the first and second time point (see Figure S1), although the reduction was significant only in the cases of Ukomba, Arena Blanca, and Cogo. The number of effective alleles (*A_e_*) over time is represented in Figure S2, and displays the same trends as the reported *H_E_*. In the six populations for which samples were collected prior/close to the start of the vector control, only in *An. gambiae* (M) from Ukomba was a slight (non-significant) decline in *H_E_* detected coincidental with the vector control. The two Bioko Island populations, *An. gambiae* (M) in Punta Europa and *An. melas* in Arena Blanca, both had lower levels of *H_E_* compared to mainland values (0.65 vs. 0.70 and 0.45 vs 0.64 respectively), although the difference was substantial only in *An. melas*. There were no significant decreases in *H_E_* or *A_R_* between time points in either of the two control populations (Table 3).

**Table 2:** Summary of heterozyosity estimates (*H_E_*) and Allelic Richness (*A_R_*) for all sampled species, populations, time points, including standard errors (SE).

Species	Site	Year	N	HEXP	SE	*P_(EL)_*	*A_R_*	SE	*P_(EL)_*
***An. gambiae (M)***	Punta Europa	April, 2004	119	0.654	0.040		8.12	0.46	
		September, 2006	63	0.648	0.033	0.56	7.79	0.51	**0.26**
		August, 2007	78	0.678	0.038		7.69	0.55	
		April, 2010	94	0.631	0.039	0.96	7.27	0.41	**<0.001**
	Ukomba	March, 2007	78	0.736	0.038		9.48	0.97	
		May, 2009	46	0.681	0.037		7.98	0.51	**0.042**
		February, 2010	95	0.673	0.041	0.75	7.07	0.55	**0.001**
***An. gambiae (S)***	Mongomo	February, 2007*	83	0.700	0.043		9.02	0.67	
		April, 2009	56	0.721	0.059	0.12	9.25	1.18	**0.053**
		May, 2010	53	0.734	0.033	0.07	8.28	0.86	0.119
	Yengue	February, 2007	62	0.734	0.040		8.35	0.82	
		May, 2009	92	0.715	0.043		7.52	0.67	**0.069**
		May, 2010	32	0.743	0.037	0.53	8.19	0.58	0.387
***An. moucheti***	Neifang	May, 2007	48	0.805	0.014		10.79	0.75	
		August, 2009	34	0.845	0.011	0.36	10.19	0.82	0.184
***An. melas***	Arena Blanca	April, 2009	89	0.484	0.066		4.8	0.6	
		September, 2010	68	0.425	0.073	0.74	3.97	0.59	**0.040**
	Cogo	April, 2007	70	0.649	0.069		9.98	1.89	
		June, 2009	76	0.641	0.070	0.87	9.4	1.82	0.005
		June, 2010	50	0.644	0.069	0.94	9.78	1.86	0.253

*P* values for tests of the null hypothesis that mean heterozygosity or allelic richness is the same between time points (based on a paired t-test) are also presented. Two *P_(EL)_* values are reported for the pairwise comparison between the earliest and second, as well as the earliest and latest time points. P-values for comparisons that showed significant differences are in bold.

* The February 2007 sample is from the nearby village of Mongomoyen.

**Table 3:** Summary of heterozygosity estimates (*H_E_*) and Allelic Richness (*A_R_*) for the two negative control populations—each with two time points, and standard errors (SE).

Species	Site	Year	N	HEXP	SE	*P_(EL)_*	*A_R_*	SE	*P_(EL)_*
***An. gambiae (M)***	Tiko, Cameroon	September, 2003	52	0.739	0.028		8.6	0.70	
		August, 2006	52	0.711	0.044	0.44	8.9	0.92	0.51
	Fanzana, Mali	August, 2002	43	0.803	0.031		13.00	1.193	
		September, 2006	89	0.801	0.031	0.78	15.417	1.264	0.30

*P* values for tests of the null hypothesis that mean heterozygosity or allelic richness is the same between time points (based on a paired t-test) are also presented. One *P_(EL)_* value is reported for the pairwise comparison for statistical difference between estimates for the two time points. P-values for comparisons that showed significant differences are emboldened.

**Figure 2:**
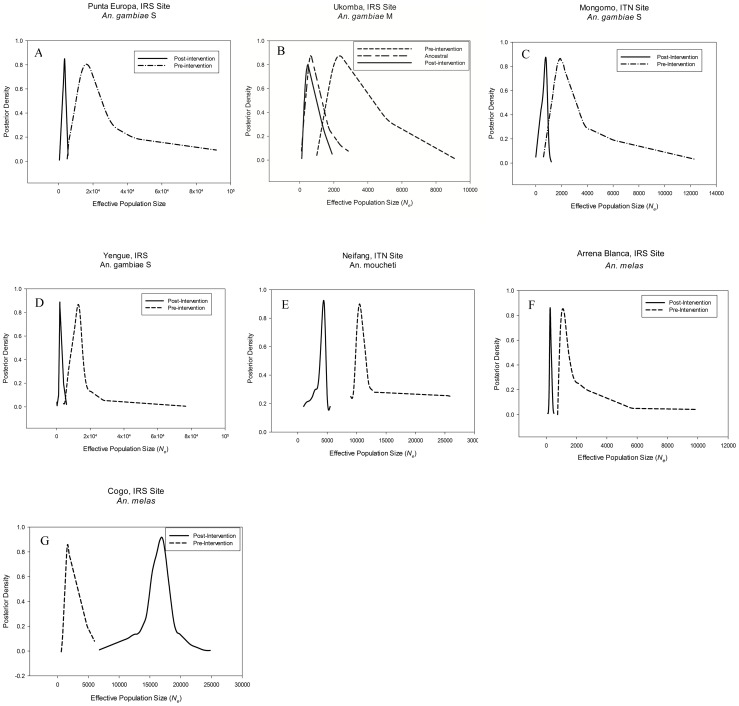
Density plots of effective population size estimates. Posterior density plots of estimated *N_e_* from ABC analysis for the seven study populations A) Punta Europa, B) Ukomba, C) Mongomo, D)Yengue, E) Niefang, F) Arena Blanca and G) Cogo. Solid line depicts the post-intervention *N_e_*, whereas the dashed line corresponds to the pre-intervention *N_e_*.

**Figure 3:**
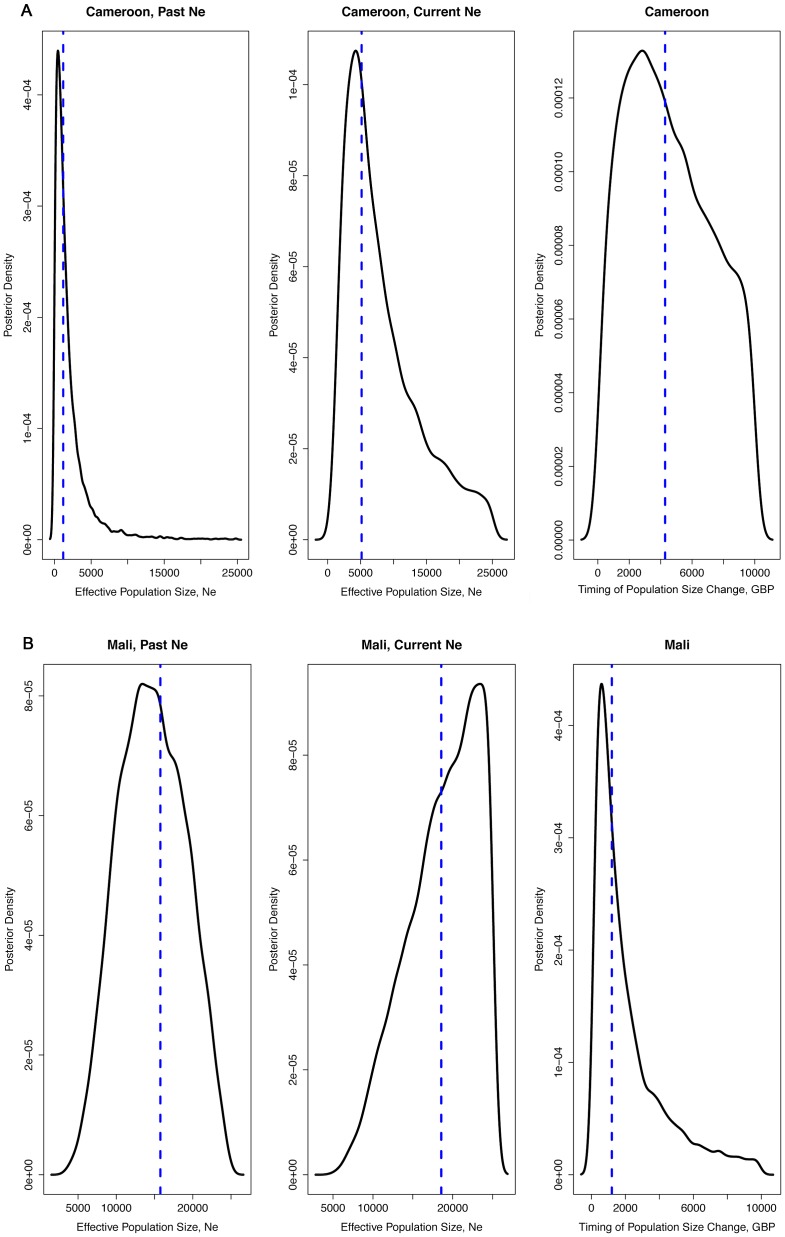
Density plots of effective population size estimates and time of population size change. Posterior density plots of estimated *N_e_* from the ABC analysis of the two negative control populations, Tiko, Cameroon (3A) and Fanzana, Mali (3B).

To infer the demographic history of each population, the microsatellite data sets were compared to ones simulated under competing demographic scenarios using ABC analyses (Figure 2, Figures S3 and S4, Table 4). Posterior density plots of *N_e_* estimates before and after control initiatives are shown in Figure 2, and posterior probability plots for the various scenarios are shown in Figure S3. The posterior density plots of the timing of demographic events are shown in Figure S4. In all but one study population (Cogo, *An.melas*), a demographic model that includes a recent decline/bottleneck that resulted in a smaller current *N_e_* provided the best fit to the observed data (Figure S3). *N_e_* typically decreased between 55% to 85% following initiation of vector control. The sole exception was *An. melas* in Cogo, in which a large historic increase in *N_e_* was detected. In contrast, neither of the negative control populations showed drastic decreases or increases over the time-scale on which vector control effects are being evaluated (Figure 3A and 3B, Table 5), but instead experienced moderate increases over much longer timescales.

**Table 4:** Results from ABC analysis for each sampled population—showing best-fit scenario, pre-intervention *N_e_* (with 0.025 and 0.975 quantiles), post intervention *N_e_* (with quantiles), and the timing of a population change (generations before present), t (with quantiles), and percentage change in *N_e_*. Medians are reported for all estimates.

Species	Population	Best-fit Scenario	Pre-intervention Ne			Post-intervention Ne				Timing		
			Ne	Q 0.025	Q 0.975	Ne	Q 0.025	Q 0.975	Change %	t	Q 0.025	Q 0.975
*An.gambiae (M)*	Punta Europa	Bottleneck (p.pr. = 0.74)	15700	6400	92100	3230	1090	4780	79	314	96	943
	Ukomba	Fluctuating (p.pr. = 0.81)	938^1^	100	6270	2570	765	15300	42	74	71	146
			13000^2^	4790	19600				83	958	268	1970
*An.gambiae (S)*	Mongomo	Bottleneck (p.pr. = 0.69)	1770	766	12300	750	468	851	57	57	11	549
	Yengue	Bottleneck (p.pr. = 0.98)	13200	4160	76600	1900	310	5190	85	88	15	326
*An.moucheti*	Neifang	Bottleneck (p.pr. = 0.87)	10300	10100	26400	4600	2510	4985	55	80	60	196
*An. melas*	Cogo	Increasing (p.pr. = 0.99)	1510	769	5810	17100	6940	24600	1132	Not estimable		
	Arena Blanca	Bottleneck (p.pr. = 0.99)	1090	801	9780	261	153	396	76	61	53	193

1 *N_e_* ancestral estimate,

2 *N_e_* historical estimate.

**Table 5:** Results from ABC analysis for the two negative control populations—showing best-fit scenario, historical *N_e_* (with 0.025 and 0.975 quantiles), recent *N_e_* (with quantiles), and the timing of a population change (generations before present), t (with quantiles), and percentage change in *N_e_*. Medians are reported for all estimates.

Species	Population	Best-fit Scenario	Historical Ne			Current Ne				Timing		
			Ne	Q 0.025	Q 0.975	Ne	Q 0.025	Q 0.975	Change %	t	Q 0.025	Q 0.975
*An.gambiae (M)*	Tiko, Cameroon	Constant/Increasing (p.pr. = 0.92)	1458	435	3570	5102	958	11980	3 Fold	4120	780	7790
	Fanzana, Mali	Constant/Increasing (p.pr. = 0.85)	15700	2850	21310	18560	7530	23950	17%	1250	325	5990

### Study Populations

#### *An. gambiae* (M), Punta Europa, Bioko Island

In the ABC analyses, the “bottleneck” scenario was the superior model (Figure S3), with a posterior probability of 0.74 (Table 4). In this area, the IRS intervention has resulted in an approximate 79% reduction in the *N_e_* of *An. gambiae* (M). The historical *N_e_* was estimated to be around 15,700 and was reduced to a current *N_e_* of approximately 3,230 (Table 4, Figure 2A). The timing of the bottleneck is estimated to be around 314 generations before the most recent collection (generations before present, gbp) (Table 4, Figure S4). However the posterior density profile for this parameter is fairly broad, meaning that this estimate has a wide credibility interval. Assuming 24 generations a year the anti-vector intervention started approximately 150 gbp. This falls well within the range of the estimate, indicating that the *N_e_* reduction occurred around the time that the intervention was implemented.

#### *An. gambiae* (M), Ukomba

A fluctuating population model, where a population expansion was followed by a more recent bottleneck provided the best fit for the data for Ukomba, with a posterior probability of 0.81 (Table 4, Figure S3). The *An. gambiae* (M) population in Ukomba had a relatively small (ancestral) population of only 938, which expanded to a larger historical size of 13,000. This was followed by a more recent, approximately 83% reduction, resulting in a current *N_e_* of approximately 2,570 (Table 4, Figure 2B). The timing of this bottleneck was estimated at 74 gbp, corresponding well with the implementation of IRS on the mainland in 2007, approximately 72 gbp. The estimate for the historical expansion had a median value of 423 gbp. Assuming 24 generations per year, the population expansion occurred about 18 years ago (1992) (Table 4, Figure S4).

#### *An. gambiae* (S), Mongomo

According to our analyses the *An. gambiae* (S) populations in Mongomo also experienced a recent bottleneck (posterior probability = 0.69, Table 4, Figure S3). The *Ne* prior to the intervention is estimated to be 1,770 but was reduced approximately 57% to a current *N_e_* of 750 (Table 4, Figure 2C). The 95% credibility interval for the timing estimate was broad, but the median value of 57 gbp corresponds closely with the start of the LLIN campaign 56 gbp (Table 4, Figure S4).

#### *An. gambiae* (S), Yengue

The bottleneck model was also the best supported for the Yengue population data, with a posterior probability of 0.98 (Table 4, Figure S3). This population experienced the greatest decline in *Ne* observed in our study. *N_e_* dropped approximately 85% from 13,200 to 1,900 (Table 4, Figure 2D). This bottleneck was estimated to have taken place at approximately 88 gbp (Table 4, Figure S4), corresponding closely to the stasrt of IRS in this location approximately 72 gbp.

#### *An. moucheti*, Niefang

The bottleneck model provided the best fit to the data from *An. moucheti* populations from Niefang, with a posterior probability of 0.87 (Table 4, Figure S3). The *An. moucheti* population at Niefang declined approximately 55% from a pre-intervention *N_e_* of about 10,300 to a current *N_e_* of 4,600 (Table 4, Figure 2E). The 95% credibility interval for the timing estimate was also broad with a median of 80 gbp (Table 4, Figure S4), and the start of the LLIN distribution approximately 60 generations ago falls well within the range of the estimate.

#### *An. melas*, Arena Blanca, Bioko Island

The *An. melas* population at Arena Blanca on Bioko Island also experienced a sharp recent reduction in *N_e_*, with the bottleneck model having a posterior probability of 0.99 (Table 4, Figure S3). The *N_e_* of *An. melas* in this location dropped approximately 76%, from 1,090 to 261 (Table 4, Figure 2E). The estimate of timing of this reduction is approximately 61 gbp (Table 4, Figure S4), and the initiation of IRS on Bioko Island approximately 140 gbp ago falls at the upper limit of the range of the estimate.

#### *An. melas*, Cogo

The *An. melas* population in Cogo was the only one not experiencing a reduction in *N_e_* coinciding with the start of the intervention. In fact, a demographic model describing a dramatic increase in *N_e_* provided the best fit to the data, with a posterior probability of 0.99. (Table 4, Figure S3). The *An. melas* population at this site increased from a historic *N_e_* estimate of 1,510 to a current *N_e_* of 17,100 (Table 4, Figure 2G). Unfortunately, no estimate of the timing of this event was possible, as the posterior probability distribution is completely flat across the range of the priors (5000 generations, not shown).

### Negative Control Populations

#### *An.gambiae* (M), Tiko, Cameroon

A demographic model describing a constant or increasing population size was the best-fit model (posterior probability = 0.92) for the negative control population in Tiko. *N_e_* estimates indicate that it increased from a historic *N_e_* of 1,458 to a current *N_e_* of 5,102 (Table 5, Figure 3A). This expansion is estimated to have occurred approximately 4,120 gbp (about 160 years ago).

#### *An.gambiae* (M), Fanzana, Mali

As with Tiko, Cameroon, the demography of the negative control population at Fanzana, Mali was also best described by a constant/increasing population model (posterior probability = 0.85). In this case, a historic population with *N_e_* = 15,700 increased moderately (17%) to a present *N_e_* of 18,560 (Table 5, Figure 3B). This change in population size was estimated to have occurred approximately 1,250 gbp (about 50 years ago).

### Confidence in Scenario Choice

We performed an analysis of the confidence in the choice of the best scenario using the simulation approach implemented in DIY ABC. The type I error (probability of falsely rejecting the correct hypothesis) for each population ranged from 6.6–27.6% (Table S1). More important for evaluating our conclusions is the probability of the inferred scenario being the incorrect one (type II error). This probability is very low in each of the nine analyses, ranging between 0.8% and 3.8% (Table S1). These low type II error estimates strongly support our conclusions regarding the inferred demographic scenarios for all nine populations, and the rejection of alternate scenarios.

## Discussion

Over the last few years ABC approaches have emerged as powerful analytical tools for exploring demographic history in fields as varied as conservation biology, epidemiology, and phylogeography [23], [31]. Here, we use the coalescent ABC approach to evaluate the impact of anti-vector interventions on the effective population size of three African malaria mosquitoes. Our study is the first to conclusively correlate anti-vector interventions (IRS or LLINs) with a reduction of *N_e_* in malaria mosquito populations. In all but a single case, a model describing a recent decline in population size provides the best fit for the data. In all of these six cases, the start of the intervention lies well within the boundaries of the estimate of the timing of the *N_e_* reduction. Both IRS and LLIN interventions resulted in a lowered *N_e_* compared to pre-intervention times.

The second important result is that the two types of interventions have similar consequences for vector population size. Although IRS is specifically designed to kill mosquitoes, much of its protective effect may be due to the repellent effect of the insecticide used. Although a repellent effect may still play a role in the effectiveness of IRS, clearly IRS is effective in reducing the population size of *An. gambiae* (M+S), as well as the *An. melas* population on Bioko Island. Similarly, much of the protective effect of ITNs/LLINs might also be the result of the reduction of contact between the vector and the human host as pyrethroid insecticide are known to have a repellent effect [32]. Epidemiologically, there is mixed evidence for the protective effect of IRS as compared to ITNs/LLINs, and it has been suggested that quantitative comparisons between IRS and ITNs may not be possible with present data [33].

While we did not evaluate the impacts of either vector control method on infection rates, we demonstrated that both IRS and LLINs approaches are successful in suppressing mosquito population sizes. The analysis shows that the effective population size of the anthropophilic and endophilic species *An. gambiae* (S) and *An. moucheti* can be reduced over 50% by the use of LLINs. Although the comparison is limited, the two *An. gambiae* populations controlled only by IRS experienced an 83–85% decline in *N_e_*, whereas the *An. gambiae* population from Mongomo, which was targeted with LLINs, declined only 57%. If true, this pattern could reflect the fact that only pyrethroids were used in the LLIN campaign, while a rotation of insecticide classes (described below) was used in the IRS. Alternatively, it may be that IRS is somewhat more efficient at controlling mosquitoes than LLIN.

IRS on Bioko Island, following an initial spray round using a pyrethroid, has been implemented using bendiocarb, a carbamate insecticide against which mosquito populations on the island have not yet developed resistance (HJ Overgaard *et al.* submitted). On the other hand, IRS on mainland Equatorial Guinea has been implemented using a rotation between pyrethroid and carbamate insecticides. *An. gambiae* (partial) resistance against pyrethroids is widespread in the form of knockdown resistance (*kdr*), which may undermine the effect of IRS and ITN use. *Kdr* is found in two different forms in *An. gambiae*; the *L1014F* [34] and *L1014S* [35] alleles. In Yengue the combined pre-intervention frequencies of these two alleles was already 90.4% and increased only slightly to 93.5%. However, *kdr* frequencies were low in Ukomba prior to the intervention (19.7%) and increased dramatically to 97.5% after the intervention [36].

Clearly *An. gambiae* mosquitoes carrying *kdr* had a strong selective advantage in Ukomba, which could conceivably have led to a rebounding of a *kdr* carrying population, following the initial decrease in population size of the still mostly susceptible population. As far as we can tell, this did not happen. Together with the large *N_e_* reduction observed in the Yengue population this suggests that the presence of *kdr* does not prevent pyrethroid insecticides from being highly effective, a result also supported by recent modeling approaches [37]. One might ask if the dramatic increase in *kdr* in the Ukomba population could have biased our results. Of the markers used in this study, locus AG2H770 is located closest to the sodium channel gene that carries the *kdr* allele, about 2 Mb away. However, this locus actually showed a slight, non-significant increase in *H_E_* between 2007 and 2010, indicating that selection for *kdr* is not likely to have affected our analyses of this population through a hitchhiking effect on our markers.

The *An. gambiae* (S) population in Mongomo, which was subjected solely to pyrethroids through the distribution of LLINs also experienced a sharp decline, even though *kdr* frequencies were high before the control started (90%±13.2%) [36]. Although a decline in efficacy of pyrethroid based IRS and ITN has been reported from Benin [38], most studies have actually found only a weak association between *kdr* and resistance against pyrethroids [Eg.], [ 39], [40,41]. Our results showing a substantial, non-transient decline in this *An. gambiae* population through LLINs using pyrethroids are consistent with these observations.

Interestingly, the two *An. melas* populations responded quite differently to IRS. Although *An. melas* in Arena Blanca on Bioko Island exposed to IRS had its *N_e_* impacted dramatically, this is clearly not the case in Cogo on the mainland. In that location, *An. melas* actually underwent a drastic increase from approximately 1,510 to 17,100. On Bioko Island *An. melas* readily enters houses to feed on humans [42], and this has been found in Cogo as well (MR Reddy, unpublished results). However, a study by Muirhead–Thomson [43] around Lagos, Nigeria, showed that *An. melas* is an opportunistic feeder that will feed primarily on the host that is most available. Although there are no cattle in Cogo, it is possible that this population has sufficient access to other vertebrate hosts so that only a small proportion of its blood meals come from humans, hereby making the impact of IRS negligible. In such a case, we would not expect to see a reduction in *N_e_.* This supposition is supported by the fact that *An. melas* typically has a much lower sporozoite rate than *An. gambiae s.s.* [44], [45]. In fact, none of 232 *An. melas* mosquitoes collected in Cogo in 2009 were sporozoite positive (MR Reddy, Unpublished results), whereas the sporozoite rate was 9.9% (±3.5%) in *An. gambiae* in the same location. It should be noted however that some sporozoite positive *An. melas* were collected prior to the intervention in 2007 (3.2%±1.8%) (MR Reddy, Unpublished results), showing that *An. melas* did contribute to malaria transmission to some degree in Cogo.

The lack of efficacy of IRS on the *An. melas* population in Cogo does not explain the dramatic increase in *N_e_* it experienced at some time in the past however. One possible explanation could have been that *An. melas* in Cogo was able to expand after a decline in *An. gambiae* s.s. following IRS. A reduction of *An. gambiae s.s* might have allowed *An. melas* to breed in what normally would be *An. gambiae s.s* larval habitat. Such dominance of *An. gambiae* larvae with respect to *An. arabiensis* has been demonstrated in a laboratory setting [46]. Even so, a decrease in the absolute numbers of *An. gambiae* s.s. in Kenya following ITN/LLIN use did not lead to an increase in sympatric *An. arabiensis* populations [10]. In any case, pre- and post-intervention mosquito collection data clearly indicate that this is not what happened. In 2007 the ratio of *An. melas* to *An. gambiae* s.s. in Cogo was 1.82 (n = 243) (window traps), whereas a year post-intervention this ratio declined to 1.68 (n = 284) (indoor HLC+LTC), actually indicating a small and insignificant decrease in the proportion of *An. melas.* Earlier work demonstrated large fluctuations in *An. melas* population sizes associated with rainfall and tide levels [47]; Gillies and De Meillon [4] characterized *An. melas* as a species with notoriously unstable, and very widely fluctuating numbers. However, the analysis of our negative control populations indicates that periodical expansions and contractions across a few seasons or years are not detected by our study design, and frankly we do not know the reason for this dramatic increase in effective population size of *An. melas* in Cogo.

In contrast to the Cogo populations, *An. melas* on Bioko Island clearly was affected by the IRS campaign, having its *N_e_* reduced from 1,090 to 261. This suggests that *An. melas* on Bioko Island is a more important vector than it is in Cogo. Possibly, a difference in availability of alternative hosts may play a role. For example, little livestock is being kept on Bioko Island [48], which may have resulted in *An. melas* being more closely associated with humans on the island. Alternatively, innate host preference may be different between the two populations. Deitz *et al.* [49] showed that *An. melas* in fact consists of three highly genetically divergent clusters; a Bioko Island cluster, as well as a Western and Southern cluster on the African continent.

The demographic history of *An. gambiae* (M) in Ukomba is also more complicated than what was observed in most populations. In Ukomba, a population expansion was detected that increased the *N_e_* from approx. 938 to 13,000, before once more being reduced by recent vector control. The estimate of the timing of the ancestral expansion was about 18 years ago. Other authors have provided genetic evidence for an expansion of *An. gambiae* populations in the past [50], [51]. However, these increases in population size are thought to have been associated with the expansion of *An. gambiae* across Africa and might have taken place as long as 49,000–630,000 years ago [51]. Ukomba is part of Bata, the largest city in Equatorial Guinea, and possibly changes associated with urbanization and/or the expansion of human populations in the area allowed an increase in *An. gambiae* breeding in this location during the last few decades.

How a reduction in effective population size correlates to the census population size (*N_c_*) depends on the presence and nature of larval competition. In case of strong density-dependent larval survival, it is conceivable that the reproductive output of surviving female mosquitoes increases if adult mortality is high due to IRS and LLIN use. This could conceivably lead to a reduction in *N_e_* without much of a noticeable reduction in the actual number of adult mosquitoes. However, it has been shown that an increase in larval density of *An. gambiae* s.s. leads to an extension of larval development time and smaller adult size, but has no effect on larval survival [52]. Thus the observed reduction in *N_e_* in *An. gambiae* likely represents an approximate corresponding decrease in the census population size.

As noted above, one of the major uncertainties involved in estimating mosquito numbers is how direct and indirect estimates correlate with each other. Few studies are available that compare direct and indirect methods in estimating the sample size of *Anopheles* mosquitoes. In Banambani, Mali, *An. arabiensis* population size was estimated to be between 9,073 and 36,249 using mark-release-recapture (MRR) methods [53]. This is approximately five-fold the *N_e_* of the same population, which was estimated to be between 2,230 and 5,892 using indirect methods [54]. MRR experiments in Ouagadougou, Burkina Faso, estimated population densities of *An. gambiae* s.l. to be 135,000 and 330,000 in 1991 and 1992, respectively [55]. The 1992 MRR experiment followed a period of exceptionally heavy rains, accompanied by flooding, which may explain the very large population size that year. The differences between *N_e_* and *N_c_* may be due to several factors, the most important likely being that *N_e_* is close to the minimum census size of the population during the seasonal fluctuations, whereas direct methods such as MRR measure the population size at the time the study is conducted.

These studies, while illustrating the difficulty of estimating population sizes for mosquitoes, also point to the influences of various environmental and demographic factors on mosquito populations that remain obscured, or whose impacts are not fully understood. In such cases, the ABC approach can be a useful tool to understand the demographic processes at the population level [56]. The ABC approach relaxes some of the assumptions associated with an “ideal” population and models allele frequency change based on the serial coalescent. It also allows the definition of priors for *N_e_*, which improves precision [57], and has the ability to utilize genetic summary statistics from samples that are collected on diffuse geographical and temporal scales.

The ABC approach is most powerful when samples are separated by several generations. In our study, all samples were separated by >20 generations. Within *An. gambiae*, where gene flow among populations of the same form is sufficient to preclude significant differentiation at the geographic scale of this study, the trend was comprehensive in showing substantial declines following the intervention program. This suggests that while gene flow may be sufficient to prevent population differentiation, it is not enough to augment local effective population sizes.

We did not observe a significant reduction in *H_E_* in most of the populations for which samples were collected close to the start of the campaign, with the exception of Ukomba. The lack of drop in *H_E_* is perhaps not surprising as reductions in *H_E_* can only be expected when *N_e_* is reduced to very small numbers. However, a reduction in allelic richness (*A_R_*) is expected during large population size reductions, even when the current *N_e_* is not very small, because rare alleles, which have a large impact on allelic richness, are more quickly lost during population reductions. In our study, we found that *A_R_* declined within most populations over time, although the decline was significant in only three out of the seven cases. A reduction in allelic richness was also observed in a small-scale study of *Culex quinquefasciatus* in Recife, Brazil [58]. This mosquito population was controlled through source reduction and the biological control of larvae. The study was not able to provide estimates of *N_e_*, but a small significant reduction in allelic diversity was found at a few microsatellite loci. As expected, no change in *H_E_* was observed.

Our results are consistent with field data that indicate a decreasing abundance of *Anopheles* mosquitoes and a lowered entomological inoculation rate (EIR), the most important entomological indicator of the force of transmission. EIR is the number of infective bites a person receives on average in a single day (or year), and equals the number of malaria vectors biting a single human per day (or year) multiplied by the sporozoite rate, i.e. the proportion of infective mosquitoes. Thus the force of transmission is linearly correlated with the population size of malaria vectors, and a reduction of 85% in the population size of a malaria vector would result in an 85% reduction in the EIR. Further, as a direct correlation exists between the size of a population and its vectorial capacity, the vectorial capacity would be reduced by 85% as well. Therefore, our results indicate that based on its effect on population size alone, IRS and ITN have led to an approximately 57% to 85% reduction in malaria transmission in six of the seven vector populations examined. Because IRS and LLINs can also reduce transmission by decreasing contact between the vector and the human host and by reducing vector longevity, the actual reduction in transmission by IRS and ITN could be larger.

Here we demonstrate for the first time, that IRS or LLIN interventions have a substantial impact on the effective population sizes of several species of malaria vectors. The results presented here are a testament to the inroads that vector control efforts have made in reducing the burden of malaria in Equatorial Guinea. The presence of the insecticide resistance allele *kdr* did not protect the *An. gambiae* population against a sharp reduction in *N_e_* during an ITN distribution program that relied on pyrethroid insecticides. IRS and LLIN approaches to vector control are expected to be most effective against endophagic and endophilic mosquitoes, and the effect on other vector species will vary depending on their host and resting preferences. Other vector control programs across sub-Saharan Africa would shed light on this issue by implementation of a similar study to evaluate the efficacy and status of vector populations.

## Materials and Methods

### Vector Control

Anti-vector interventions in the form of IRS started on Bioko Island in 2004 as part of the Bioko Island Malaria Control Project (BIMCP), in cooperation with the National Malaria Control Program within the Equatoguinean Ministry of Health and Social Welfare and implemented by Medical Care Development International Inc with financing from Marathon Oil Corporation, its private partners and the Government of Equatorial Guinea. Over 80% of domiciliary structures were initially sprayed with the pyrethroid-class insecticides Deltamethrin and Fendon during an initial spray round in 2004. After high levels of *kdr*, a target site mutation conferring resistance to pyrethroids and DDT was detected in the course of routine monitoring [59], [60], Deltamethrin and Fendon were replaced with Ficam (bendiocarb) starting in 2005, a carbamate insecticide compound, and spraying frequency was increased to two rounds per year.

Based on the success on Bioko Island, the control efforts were expanded to mainland Equatorial Guinea in 2007 under the Equatorial Guinea Malaria Control Initiative (EGMCI), funded by the Global Fund to Fight AIDS, Tuberculosis, and Malaria. Whereas a majority of control efforts on Bioko Island have been achieved through continuous IRS rounds, supplemented by an island-wide LLIN door-to-door distribution and hang-up campaign in 2008, sites on mainland Equatorial Guinea have received either IRS or LLINs. In LLIN areas, bed-nets impregnated with pyrethroid insecticide were distributed to all households through a similar door-to-door strategy. In IRS areas a rotating combination of Ficam, Alphycypermetrhin, and Deltamethrin were employed. This use of two control methods provides an opportunity to determine the relative impacts of these two approaches on mosquito populations.

### Study Sites

The study was conducted across several sites in Equatorial Guinea (Figure 1), with two additional negative control sites outside Equatorial Guinea (details below). *An. gambiae* (M) was sampled in two sites; Punta Europa on Bioko Island and Ukomba on the mainland. The Punta Europa area of Bioko Island is located to the west of the capital Malabo, and consists of three small villages with approximately 80 houses. Punta Europa is also the base of Marathon Oil Corporation and partners' industrial operations on Bioko. This compound houses their largely expatriate personnel onsite. *An. gambiae* (M) is currently the dominant vector in Punta Europa and has been targeted by IRS since April 2004. Ukomba is a suburb of Bata, the largest city in continental Equatorial Guinea with an estimated population of approximately 175,000 in 2005.

*An. gambiae* (S) was sampled in two mainland sites: Mongomo and Yengue. Mongomo is on the eastern border of continental Equatorial Guinea. It is a city consisting of approximately 6,000 houses, and one of two study sites where vector control was implemented through LLIN distribution. For Mongomo, we combined samples from a nearby location (Mongomoyen, ∼15 km distant) as no significant genetic structuring has been found within the molecular forms of *An. gambiae* on this geographical scale [61], [62]. In any case, an analysis without the Mongomoyen samples did not substantially alter the result. Yengue is a small village consisting of approximately 50 houses in the Northwest of the Equatoguinean mainland. Unlike other study sites, Yengue received only a single round of IRS at the start of the campaign in 2007.

*An. melas* was sampled from one site on Bioko Island, Arena Blanca, as well as a mainland site, Cogo. Arena Blanca is a small town on the western coast of Bioko Island with approximately 40 houses and is a popular beach location for Bioko Islanders. *An. melas* is the dominant vector in this location. Cogo is a small town located in the Southwest of the mainland containing approximately 400 houses. It is located on the Rio Muni estuary, where the brackish water provides ideal breeding sites for *An. melas*. Finally, *An. moucheti* was examined from a single location on the mainland, Niefang. Niefang is a small town consisting of approximately 2,000 houses, and is the second study location where vector intervention consisted of LLIN distribution.

Sites were selected based on the availability of samples from multiple time points. The locations, time points and species sampled are presented in Table 1. A total of 1,519 mosquitoes were analyzed in this study and sample size ranged from 32 to 95 for each time point, the average being 76. We included data sets from two negative control populations without a history of anti-vector interventions. The first was comprised of data for 11 microstallites for *An. gambiae* (M) samples from Tiko, Cameroon for the years 2002 (N = 52) and 2006 (N = 52). The second represented 11 microsatellite loci for *An. gambiae* (M) from Fanzana, Mali for the years 2003 (N = 43) and 2006 (N = 89). These data were obtained from the public population genetic database on malaria mosquito populations available from the University of California – Davis (https://grassi2.ucdavis.edu/PopulationData/). These negative control data sets were analyzed using the same approach as applied to the study populations.

### Genetic Analysis

DNA was extracted from the abdomens of individual mosquitoes preserved in 70% ethanol on a Qiagen Biosprint (Qiagen Inc, Valencia, CA) automated DNA Isolation platform and resuspended in 200 µl elution buffer. Individual locus PCRs were carried out in 20 µl reactions with 10 mM dNTP, 2 mM MgCl2, 1 µmol fluorescently labeled (6-FAM, NED, or HEX) forward primer and 1 µmol reverse primer, in a Promega reagent master mix with GoTaq Flexi Polymerase and 5× buffer. All *An. gambiae* (M & S) were genotyped at 17 microsatellite loci that were developed for this species [63]. *An. melas* was genotyped at 15 loci that were adapted for the species [64] based on the loci published by Zheng *et al.* [63], whereas 13 loci were genotyped for *An. moucheti* [65]. After successful amplification was confirmed by gel electrophoresis-visualization of random samples, samples were prepared for fragment analysis with 1 µl of ROX 500-HD size standard (Applied Biosystems, Foster City, CA), and analyzed on ABI 3730× 96-capillary analyzers at the DNA sequencing facility on Science Hill (Yale University, New Haven, CT).

### Data Analysis

Raw data was downloaded into Genemarker (Softgenetics, State College, PA), and alleles were called using panels created for each locus, using bins with a width of 1 bp. Allelic data for each locus was imported into the MS Excel based genetic analysis software GENALEX 6 [66], and formatted for further analysis. Once genotypes were recorded, the data were checked for failed amplification, presence of null alleles and large allele dropout using the program MICROCHECKER [67]. Samples with possible null alleles were re-genotyped or discarded. Following tests of HW-equilibrium and for the presence of null alleles, loci with a significant excess of homozygotes possibly indicating null alleles were dropped from further analysis. Final analyses were conducted on 12 to 17 loci depending on the population and species. Unbiased expected heterozygosity (*H_E_*) was calculated and compared between the earliest and second time points, as well as between the earliest and latest time points for each location using a paired t-test. Allelic richness (*A_R_*) was calculated for each population-time point combination in the genetic analysis software FSTAT. *A_R_* is expected to be more sensitive than *H_E_* to sudden declines in population sizes, as allelic diversity is impacted by the removal of rare alleles, which hardly affects heterozygosity. Calculations of means, standard errors and statistical comparisons for *H_E_* and *A_R_* were performed in R (version 2.14.1), running on RStudio (version 0.95).

To examine changes in *N_e_* over time, Approximate Bayesian Computation (ABC) as implemented in the program DIY ABC [68], [69] was used. ABC [22] is a coalescent statistical method that utilizes summary statistics from one or more observed population samples, and compares it with data simulated from hypothetical scenarios to find the scenario that best explains the observed data. First, a large number of datasets were simulated based on multiple user-defined demographic scenarios/hypotheses, mirroring the genetic marker, the number of loci, and appropriate mutation rates and models. Each scenario contained the parameters of interest to be estimated (historical or present *N_e_* for example), values for which were drawn from uniform prior distributions during simulations. After the simulation of 1 million datasets for each scenario, Euclidean distances between observed and simulated datasets were computed, and 1% of the closest datasets were retained. Logistic regression was subsequently performed to estimate the posterior probabilities of best-fit scenarios and to estimate the parameters of interest. We selected the ABC approach over other available methods for estimating *N_e_* and demographic history because of difficulties in estimating *N_e_* from species with overlapping generations and potentially fluctuating population sizes [70], [71]. Additionally, the window trap samples that represented one month of collections, did not allow point estimators to be used. Finally, by utilizing a number of different genetic summary statistics, the ABC method is less vulnerable to violation of assumptions made in classical population genetic models, and hence is less biased [56], [72].

For each population studied, a number of hypothetical scenarios were coded to describe possible demographic histories and to explore alternate outcomes that may follow any given demographic event. Scenarios tested and priors used are summarized in Table S2. In brief, four hypotheses, with minor variations, were tested on data from each population (Figure S5) – namely a) fluctuating *N_e_*, b) increasing *N_e_*, c) population bottleneck *N_e_*, resulting in lower contemporary *N_e_* and d) constant population size. We assumed 24 generations per calendar year [73], [74]. The mutation model was set to the general mutation model for microsatellites, which includes both stepwise and infinite-allele modes of mutation. Mutation rates were either considered to vary independently by locus, or vary around the same mean value. Following the simulation of 1 million datasets, the posterior probabilities of the tested scenarios were calculated through logistic regression on 1% of the closest datasets to determine the best explanatory scenario. The scenario with the highest posterior probability was chosen to estimate the parameters of interest.

Although ‘null hypotheses’ are not typically defined in Bayesian analyses, selection of hypothetical scenarios based on posterior probability still provides opportunities for falsely rejecting the correct hypothesis (type I error), or accepting a hypothesis when it is in fact not the correct one (type II error). In order to estimate the false positive (type I) and false negative rate (type II), given the size and nature of our simulated datasets, we estimated both type I and type II errors for each of the nine populations as described in [68]. This was done by simulating 1,000 data sets assuming in turn that each of the four scenarios is the “true” scenario, determining the posterior probabilities of all four scenarios for each simulated data set, and calculating how often the assumed “true” scenario had the highest posterior probability (Table S2).

### Data Accessibility

Microsatellite data deposited in the Dryad Repository: http://dx.doi.org/10.5061/dryad.1rf75.

## Supporting Information

Figure S1 Mean Allelic Richness (*A_R_*) estimates for the seven sampled populations. Sampled times are on x-axis, and *A_R_* are on the y-axis. Error bars are standard errors.
(PDF)

Figure S2 Mean number of effective alleles (*A_e_*) for each of the seven sampled populations. Error bars are standard errors.
(PDF)

Figure S3 Posterior-probability plots comparing tested scenarios from ABC analysis, for each study population. The scenario with the highest posterior probability (y-axis) over 1% of simulated datasets (x-axis) was the best-fit scenario. This scenario was selected to estimate the posterior probabilities of estimated parameters (*N_e_*, and t).
(PDF)

Figure S4 Posterior density distributions of estimated timing (generations before present) of the bottleneck event, if population change occurred. In case of Ukomba, the two curves show the ancestral expansion event and the more recent post-intervention event. The dashed (red) line shows the approximate time when anti-vector interventions started in each location .
(PDF)

Figure S5 A schematic showing the four typical hypotheses tested on each set of sampled populations. In scenario 1, population could fluctuate from an ancestral *N_e_* (Nanc) to a historical *N_e_* (Nhist) to a post-intervention *N_e_* (Npres).
(PDF)

Table S1 Type I and Type II error probabilities for each of the seven populations in the study. Error probabilities were calculated based on simulating 1000 datasets with an assumed “true scenario”. Type I error is obtained by cumulating across the table for a given scenario, where as type II error probabilities are listed in the column under each specified scenario. Type II errors indicate the probability that the wrong scenario has the highest posterior probability in our analyses. For example, for Punta Europa the probability that our analysis resulted in scenario 3 having the highest posterior probability if scenario 1 was actually the correct one equals 3.5%. The scenario with the highest posterior probability for each study population is shaded in gray.
(DOCX)

Table S2 A description of the most common demographic models tested using Approximate Bayesian Computation (ABC) on multiple time-point samples from Anopheline samples from each site. Generation between samples was customized for each species-site combination, assuming 24 generations/year. Additional models or variants were also run based on each particular case or when one of the common models were not the substantially better than the competing scenarios.
(DOCX)
