# Supplementary figures and images for: The Effective Population Size of Malaria Mosquitoes: Large Impact of Vector Control
Source: PLoS Genet. 2012 Dec 13;8(12):e1003097. doi: 10.1371/journal.pgen.1003097 (PMC3521722; doi:10.1371/journal.pgen.1003097)

Allelic Richness (AR)

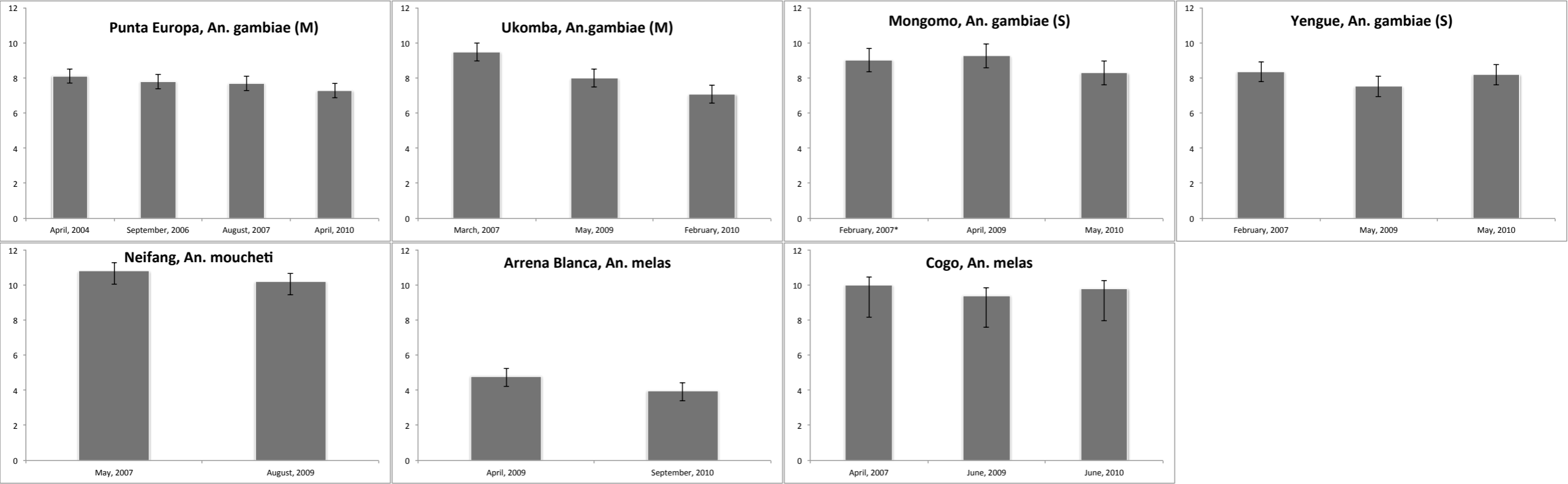

Sample Time Points

Supplement: Figure S1 — Mean Allelic Richness (AR) estimates for the seven sampled populations. Sampled times are on x-axis, and AR are on the y-axis. Error bars are standard errors. (PDF) [file pgen.1003097.s001.pdf]

# Effective Number of Alleles ( $A_e$ )

Effective Number of Alleles

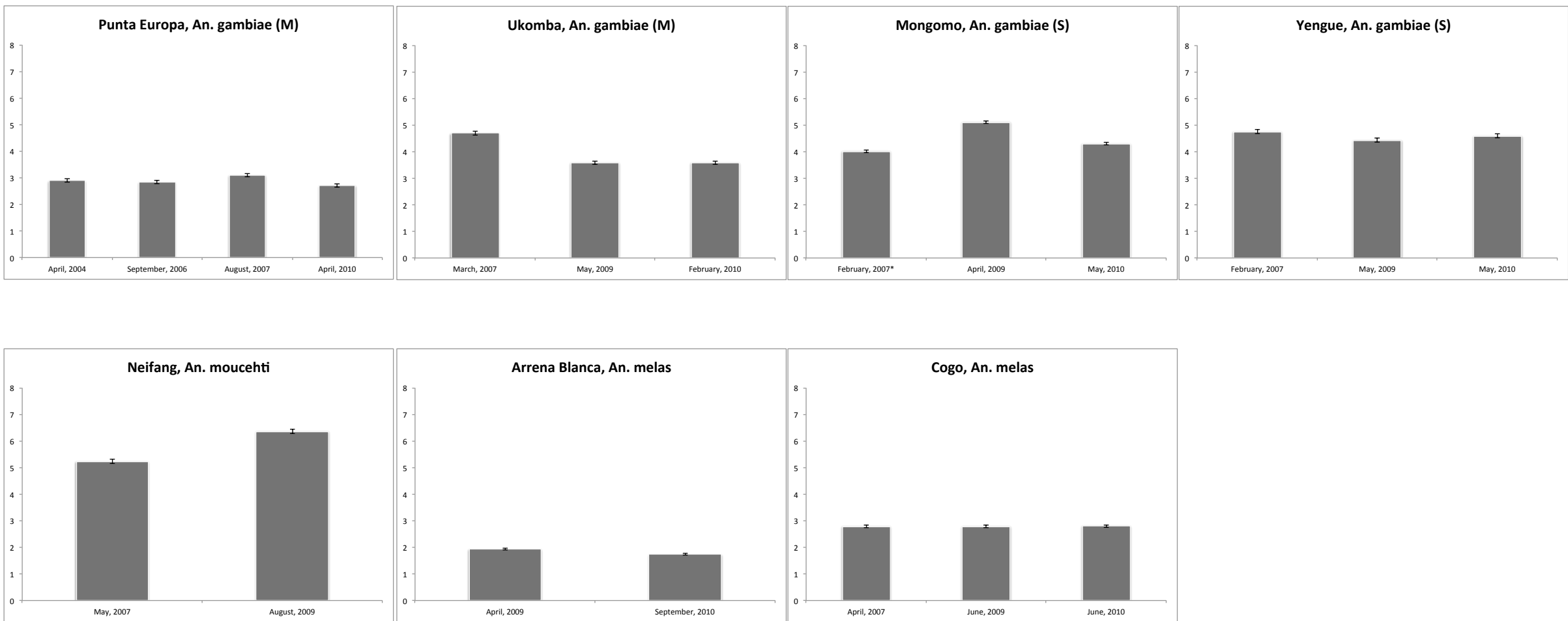

Sample Time Points

Supplement: Figure S2 — Mean number of effective alleles (Ae) for each of the seven sampled populations. Error bars are standard errors. (PDF) [file pgen.1003097.s002.pdf]

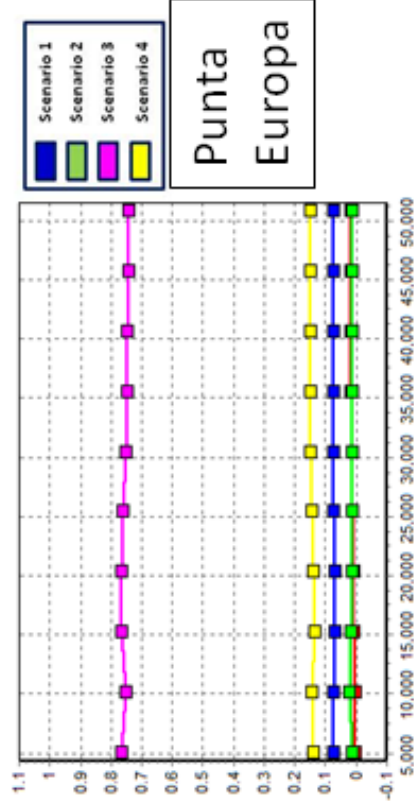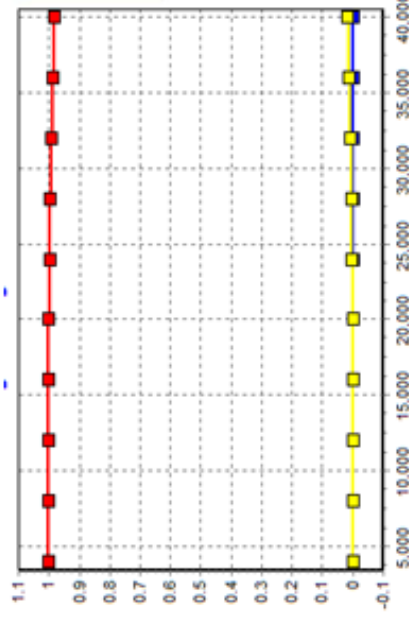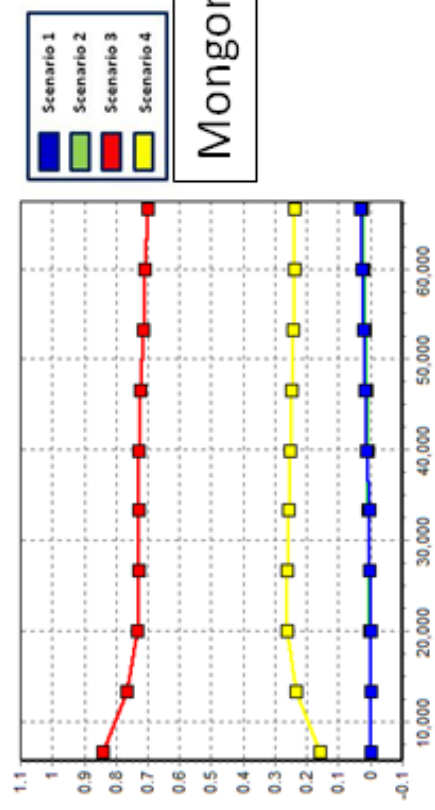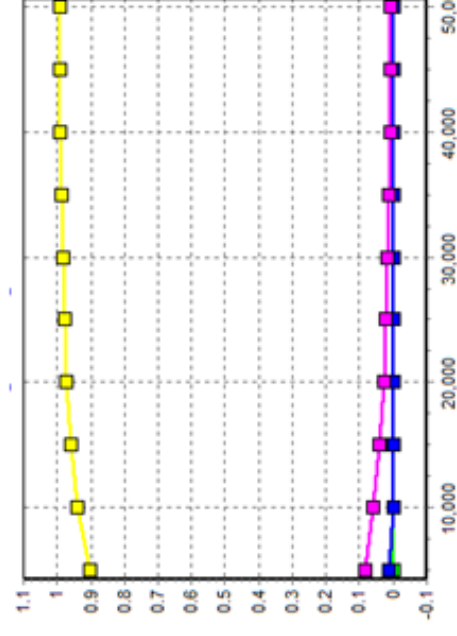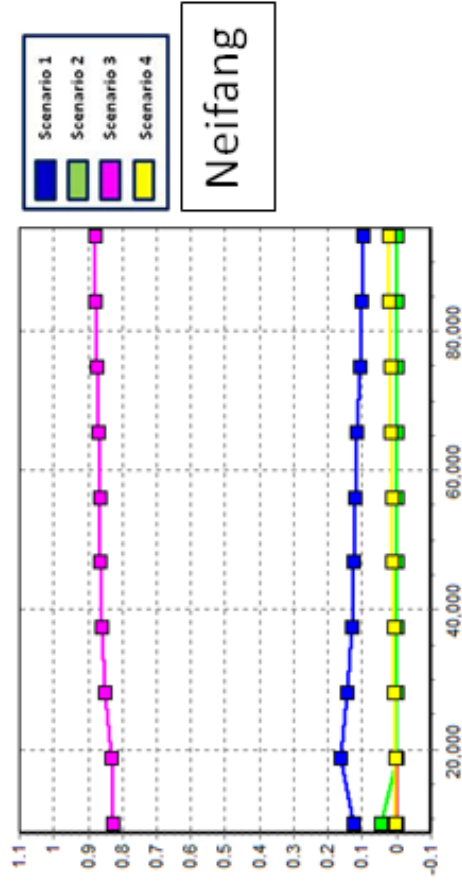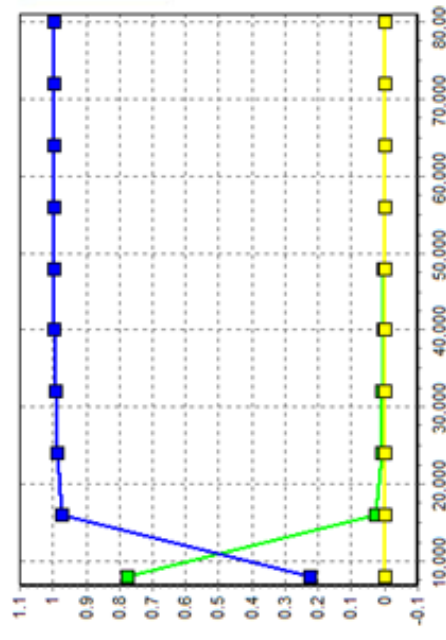

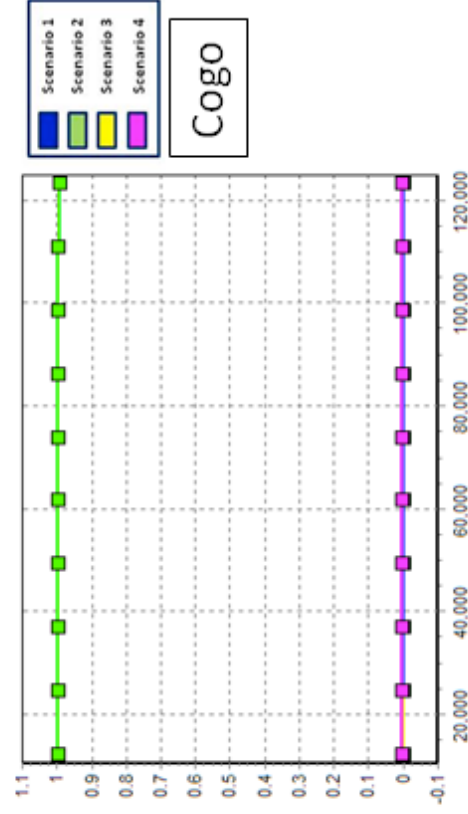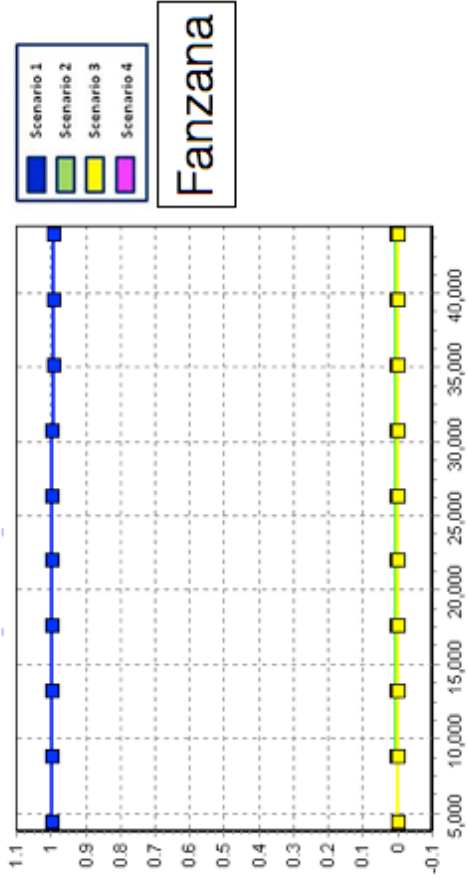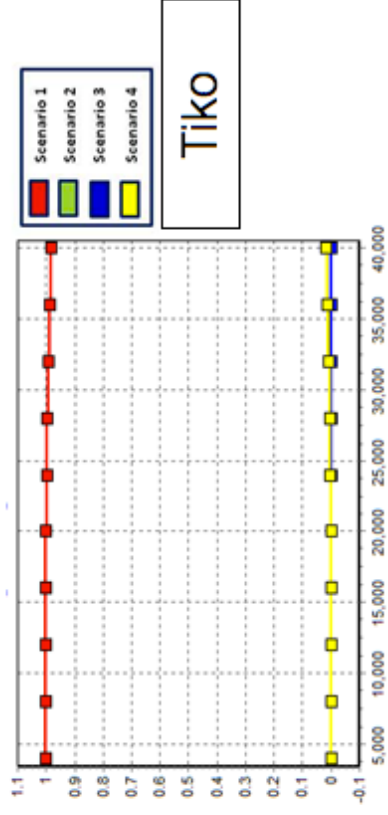

Supplement: Figure S3 — Posterior-probability plots comparing tested scenarios from ABC analysis, for each study population. The scenario with the highest posterior probability (y-axis) over 1% of simulated datasets (x-axis) was the best-fit scenario. This scenario was selected to estimate the posterior probabilities of estimated parameters (Ne, and t). (PDF) [file pgen.1003097.s003.pdf]

**Punta Europa**

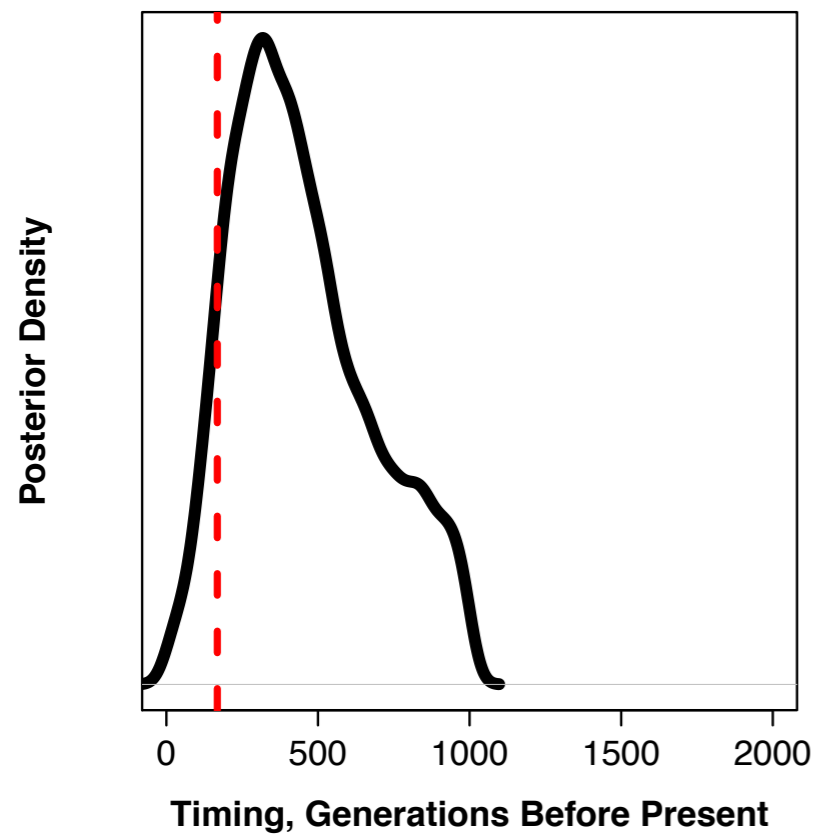

**Ukomba**

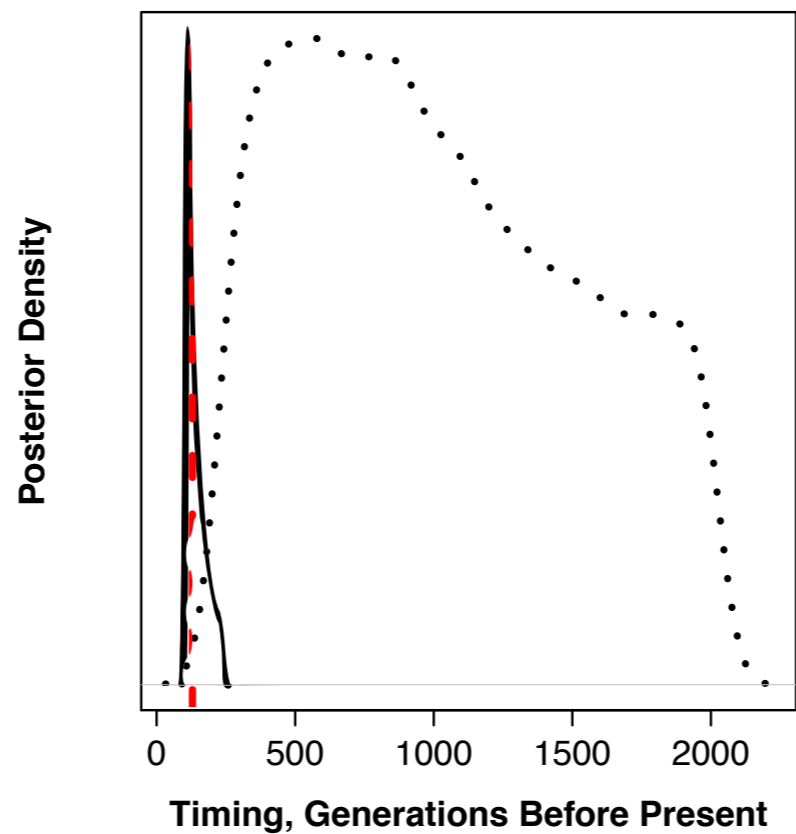

**Mongomo**

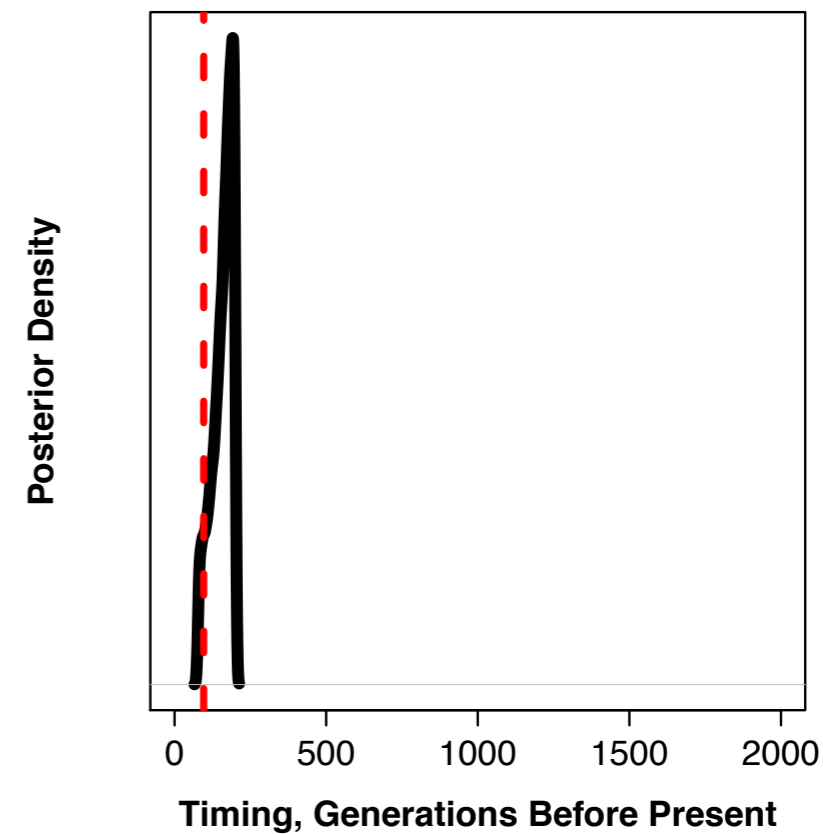

**Yengue**

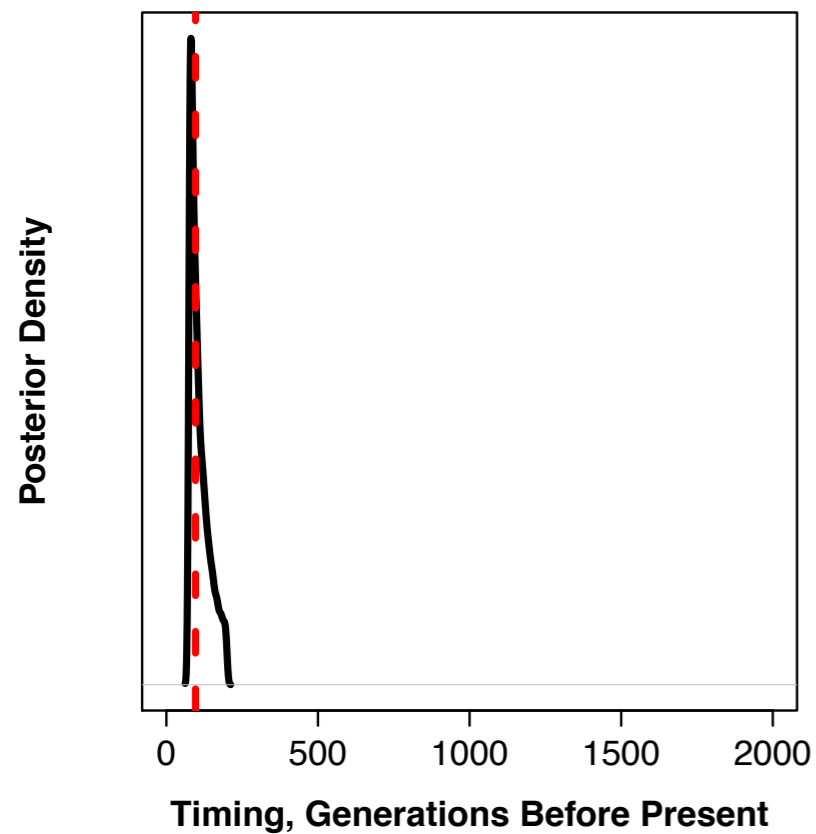

**Neifang**

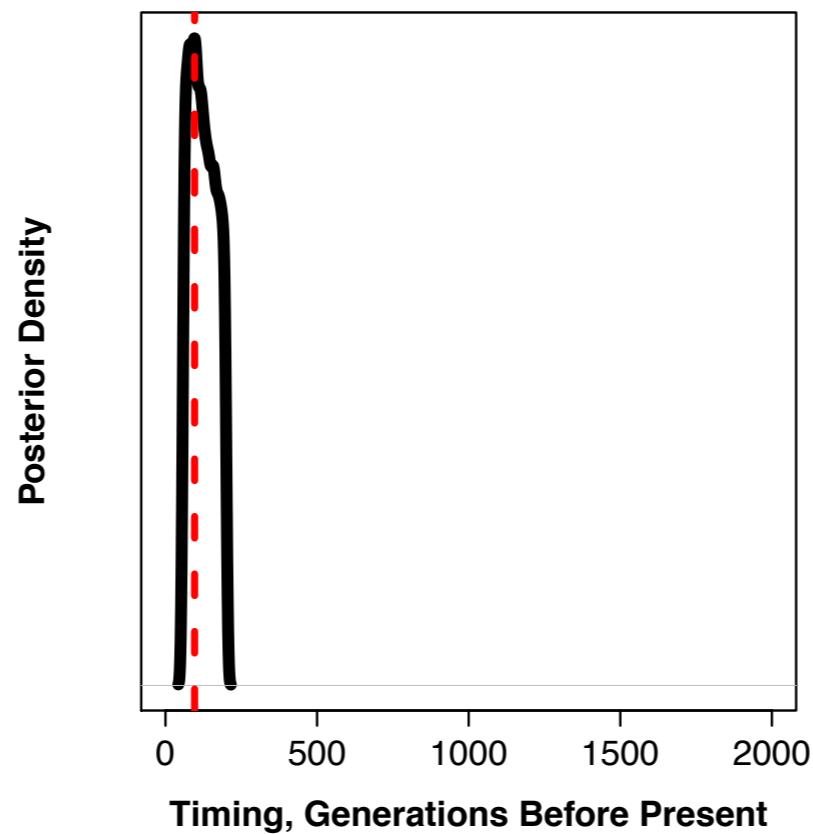

**Arrena Blanca**

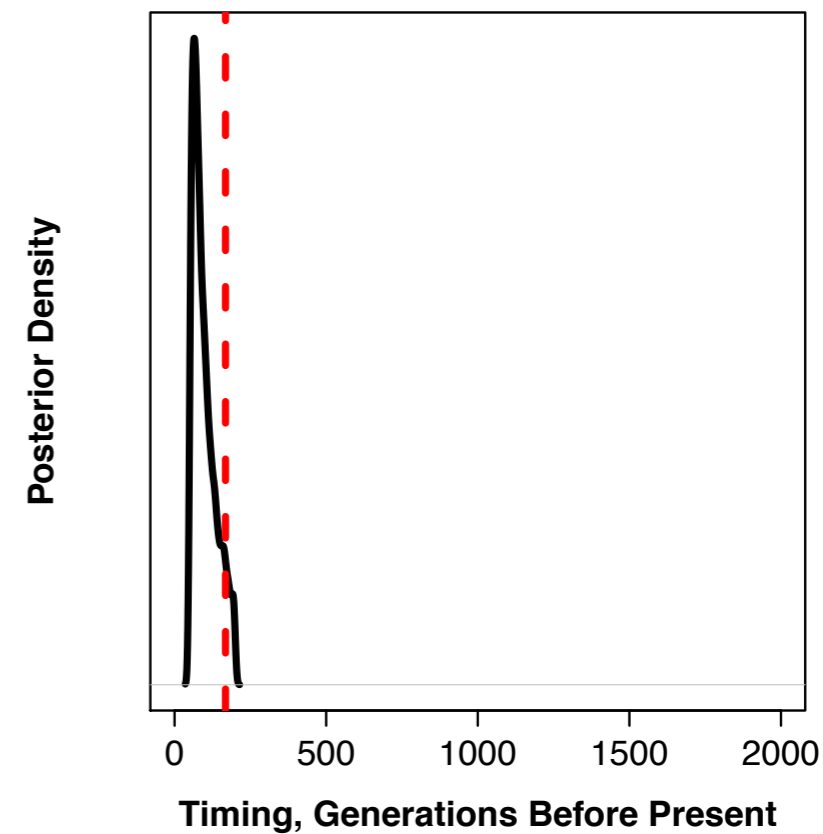

Supplement: Figure S4 — Posterior density distributions of estimated timing (generations before present) of the bottleneck event, if population change occurred. In case of Ukomba, the two curves show the ancestral expansion event and the more recent post-intervention event. The dashed (red) line shows the approximate time when anti-vector interventions started in each location . (PDF) [file pgen.1003097.s004.pdf]

# Hypothetical modeled scenarios

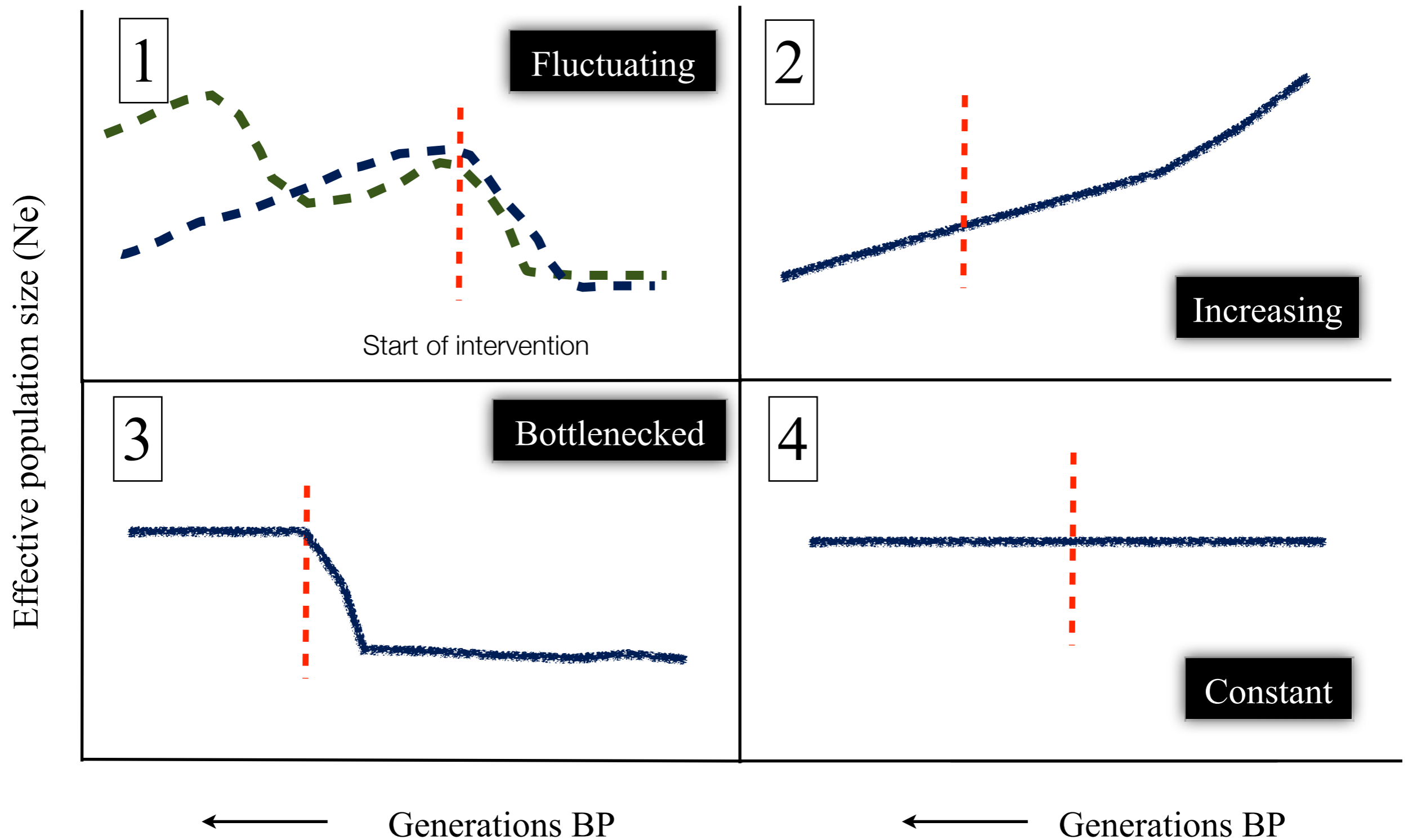

Supplement: Figure S5 — A schematic showing the four typical hypotheses tested on each set of sampled populations. In scenario 1, population could fluctuate from an ancestral Ne (Nanc) to a historical Ne (Nhist) to a post-intervention Ne (Npres). (PDF) [file pgen.1003097.s005.pdf]
