# Supplementary material for: The Effective Population Size of Malaria Mosquitoes: Large Impact of Vector Control
Source: PLoS Genet. 2012 Dec 13;8(12):e1003097. doi: 10.1371/journal.pgen.1003097 (PMC3521722; doi:10.1371/journal.pgen.1003097)
Supplement: Table S1 — Type I and Type II error probabilities for each of the seven populations in the study. Error probabilities were calculated based on simulating 1000 datasets with an assumed “true scenario”. Type I error is obtained by cumulating across the table for a given scenario, where as type II error probabilities are listed in the column under each specified scenario. Type II errors indicate the probability that the wrong scenario has the highest posterior probability in our analyses. For example, for Punta Europa the probability that our analysis resulted in scenario 3 having the highest posterior probability if scenario 1 was actually the correct one equals 3.5%. The scenario with the highest posterior probability for each study population is shaded in gray. (DOCX) [file pgen.1003097.s006.docx]

Table S1: Type I and Type II error probabilities for each of the seven populations in the study. Error probabilities were calculated based on simulating 1000 datasets with an assumed “true scenario”. Type I error is obtained by cumulating across the table for given scenario, where as type II error probabilities are listed in the column under each specified scenario. Type II errors indicate the probability that the wrong scenario has the highest posterior probability in our analyses. For example, for Punta Europa the probability that our analysis resulted in scenario 3 having the highest posterior probability if scenario 1 was actually the correct one, equals 3.5%. The scenario with the highest posterior probability for each study population is shaded in gray.

|  |  | 1 | 2 | 3 | 4 |  |
| --- | --- | --- | --- | --- | --- | --- |
|  | True scenario used for simulation | Type II Error | | | | Type I Error |
|  | 1 |  | 0.095 | **0.035** | 0.146 | 0.276 |
| Punta Europa | 2 | 0.082 |  | **0.030** | 0.080 | 0.192 |
|  | 3 | 0.037 | 0.030 |  | 0.028 | 0.095 |
|  | 4 | 0.127 | 0.077 | **0.025** |  | 0.230 |
|  |  |  |  |  |  |  |
|  | 1 |  | 0.032 | 0.032 | 0.015 | 0.079 |
| Ukomba | 2 | **0.023** |  | 0.049 | 0.089 | 0.161 |
|  | 3 | **0.028** | 0.026 |  | 0.036 | 0.090 |
|  | 4 | **0.027** | 0.071 | 0.034 |  | 0.132 |
|  |  |  |  |  |  |  |
|  | 1 | - | 0.054 | **0.018** | 0.074 | 0.146 |
| Mongomo | 2 | 0.022 | - | **0.019** | 0.084 | 0.124 |
|  | 3 | 0.036 | 0.030 | **-** | 0.029 | 0.095 |
|  | 4 | 0.070 | 0.055 | **0.026** | - | 0.151 |
|  |  |  |  |  |  |  |
|  | 1 | - | 0.032 | **0.029** | 0.047 | 0.108 |
| Neifang | 2 | 0.018 | - | **0.033** | 0.047 | 0.098 |
|  | 3 | 0.032 | 0.033 | **-** | 0.031 | 0.095 |
|  | 4 | 0.037 | 0.058 | **0.023** | - | 0.118 |
|  |  |  |  |  |  |  |
|  | 1 | - | 0.065 | **0.010** | 0.086 | 0.161 |
| Arrena Blanca | 2 | 0.006 | - | **0.008** | 0.063 | 0.076 |
|  | 3 | 0.019 | 0.023 | **-** | 0.024 | 0.066 |
|  | 4 | 0.054 | 0.030 | **0.026** | - | 0.110 |
|  |  |  |  |  |  |  |
|  | 1 | - | **0.038** | 0.042 | 0.072 | 0.152 |
| Cogo | 2 | 0.005 | **-** | 0.033 | 0.039 | 0.077 |
|  | 3 | 0.027 | **0.022** | - | 0.046 | 0.095 |
|  | 4 | 0.073 | **0.010** | 0.030 | - | 0.113 |
|  |  |  |  |  |  |  |
