# Supplementary material for: The Effective Population Size of Malaria Mosquitoes: Large Impact of Vector Control
Source: PLoS Genet. 2012 Dec 13;8(12):e1003097. doi: 10.1371/journal.pgen.1003097 (PMC3521722; doi:10.1371/journal.pgen.1003097)
Supplement: Table S2 — A description of the most common demographic models tested using Approximate Bayesian Computation (ABC) on multiple time-point samples from Anopheline samples from each site. Generation between samples was customized for each species-site combination, assuming 24 generations/year. Additional models or variants were also run based on each particular case or when one of the common models were not the substantially better than the competing scenarios. (DOCX) [file pgen.1003097.s007.docx]

Table S2: A description of the most common demographic models tested using Approximate Bayesian Computation on multiple time-point samples from Anopheline samples from each site. Generation between samples was customized for each species-site combination, assuming 24 generations/year. Additional models or variants were also run based on each particular case or when one of the common models were not the substantially better than the competing scenarios.

| **Scenario** | **Parameter** | **Description** | **Prior** | **Steps** | **Conditions enforced** |
| --- | --- | --- | --- | --- | --- |
| Fluctuating | Npres | Post-intervention Ne | U[10:100000] | 100 |  |
|  | Nhist | Pre-intervention Ne | U[10:100000] | 100 |  |
|  | Nanc | Ancestral Ne | U[10:100000] | 100 |  |
|  | t1 | time at which Nhist changed to Npres | U[1:200] | 1 |  |
|  | t2 | time at which Nanc changed to Nhist | U[200:5000] | 10 |  |
|  |  |  |  |  |  |
| Bottleneck | Npres | Post-intervention Ne | U[10:20000] | 100 |  |
|  | Nhist | Pre-intervention Ne | U[10:100000] | 100 | Nhist>Npres |
|  | t1 | time at which Nhist changed to Npres | U[1:200] | 1 |  |
| Expanding | Npres | Post-intervention Ne | U[10:100000] | 100 |  |
|  | Nhist | Pre-intervention Ne | U[10:100000] | 100 | Nhist<Npres |
|  | t1 | time at which Nhist changed to Npres | U[1:200] | 1 |  |
|  |  |  |  |  |  |
| Constant or | Npres | Post-intervention Ne | U[10:100000] | 100 |  |
| Declining | Nhist | Pre-intervention Ne | U[10:100000] | 100 | Nhist≥Npres |
|  | t1 | time at which Nhist changed to Npres | U[1:200] | 1 |  |
|  |  |  |  |  |  |
| Complex* | Npres | Post-intervention Ne | U[10:100000] | 100 |  |
|  | Nhist | Pre-intervention Ne | U[10:100000] | 100 |  |
|  | Nanc | Ancestral Ne | U[10:100000] | 100 |  |
|  | Nmid | Ne between Nhist and Npres | U[10:20000] | 100 | Nhist>Nmid, Nmid>Npres |
|  | t1 | time at which Nhist changed to Npres | U[1:200] | 1 |  |
|  | t2 | time at which Nanc changed to Nhist | U[200:5000] | 10 |  |
|  | t3 | time at which Nhist changed to Npres | U[1:200] | 1 | t1>t3 |

* was used only in cases where at least three time-point samples were available.
